# Efficacy of Curcumin Gel Versus Hyaluronic Acid as Adjuvants to Scaling and Root Planing on IL-1β Levels in Smokers With Chronic Periodontitis

**DOI:** 10.7759/cureus.101405

**Published:** 2026-01-12

**Authors:** Laxmi Jaahnavi Devarampati, Rekha R Koduganti, Veerendranath Reddy Panthula, Jammula Surya Prasanna, Hima Bindu Gireddy, Manasa Ambati

**Affiliations:** 1 Periodontics, Panineeya Institute of Dental Sciences, Hyderabad, IND

**Keywords:** chronic periodontitis, current smokers, former smokers, il-1 beta, scaling and root planing

## Abstract

Background: Periodontitis is triggered by plaque biofilm interacting with microorganisms and the host, causing destruction of the periodontal tissues and, ultimately, the loss of the tooth or teeth involved. Though scaling and root planing (SRP) is the cornerstone of periodontal intervention, it is understandable that adjuvant therapy would be beneficial in smokers with periodontitis. The use of systemic antibiotics as adjuncts in smokers has been deferred due to their innumerable side effects. This has paved the way for the administration of local drug delivery (LDD) agents. This study aimed to understand and compare the benefits of curcumin and hyaluronic acid LDD agents when used as adjuncts in smokers with periodontitis.

Material and methods: A total of 36 patients attending the outpatient Department of Periodontics from a referral care hospital, who were diagnosed clinically with chronic periodontitis and willing to participate, were included, and the study was conducted from January 2021 to April 2021. The subjects were equally divided into three groups, each group comprising 12 individuals. Group I included current smokers with chronic periodontitis (n=12) who underwent SRP followed by local drug delivery of curcumin gel in subgroup IA (n=6) and hyaluronan gel in subgroup IB (n=6). The patients in Group II were former smokers with chronic periodontitis (n=12) who underwent SRP, followed by local drug delivery of curcumin gel in subgroup IIA (n=6) and hyaluronan gel in subgroup IIB (n=6). In Group III patients with chronic periodontitis only (n=12) underwent SRP, followed by local delivery of placebo gel. The clinical parameters like Plaque Index and probing pocket depth were assessed at baseline, eight weeks, and 12 weeks after SRP, using a William's probe. The gingival crevicular fluid (GCF) samples for IL-1β levels were collected at baseline and six weeks after nonsurgical periodontal therapy.

Results: All the groups showed improvement in both clinical and biochemical parameters when a comparison was made within and between the groups, but significant results were seen only in relation to Group IIB.

Conclusion: Group II patients (former smokers with periodontitis) showed better improvements in the variables evaluated. Patients in Group III (control group) were the next to follow. However, patients in Group I (current smokers with periodontitis) did not benefit from the administration of LDD agents.

## Introduction

Dysbiosis and inflammation in periodontitis affect the supporting tissues of the teeth [1]. Globally rampant, it is a multifactorial disease wherein many risk factors play a role in its progression, predominantly bacterial colonization, age, gender, socioeconomic status, certain systemic conditions, and smoking [2]. Cigarette smoke contains at least 400 potentially toxic substances, including hydrogen cyanide, carbon monoxide, free radicals, nicotine, nitrosamines, and a variety of oxidant gases. Tobacco use as a modifiable risk factor plays an important role in disease progression and treatment outcomes. Nicotine, which is the major constituent present in cigarettes, has many detrimental effects on the body and the periodontium. Chronic inflammation of the tissues is affected by various inflammatory mediators. Interleukin-1β (IL-1β) induces bone resorption by proteinases, and systemic disease is modulated by the release of tumour necrosis factor alpha (TNF-α), IL-1β, and IL-6. The selection of the ideal antimicrobial agent plays a vital role in the success of periodontal therapy [3]. Systemic antimicrobial therapy has been a tested treatment protocol for periodontitis. However, side effects related to their administration have paved the way for local drug delivery (LDD) agents. Curcumin is a herb that is well known for its medicinal properties. Due to its anti-inflammatory, antimicrobial, and antioxidant properties, it is being used as a host modulatory agent in periodontitis [4]. Hyaluronic acid (HA), a component of the extracellular matrix, plays a major role in tissue hydrodynamics, cell migration, and proliferation. Fibroblasts produce HA in the presence of exotoxins, and HA inhibits tissue destruction and promotes healing [5]. Hence, this study, conducted in smokers with periodontitis, evaluated the role of scaling and root planing (SRP) and the effectiveness of the test gel formulations when used as adjuvants.

## Materials and methods

**Figure 1:**
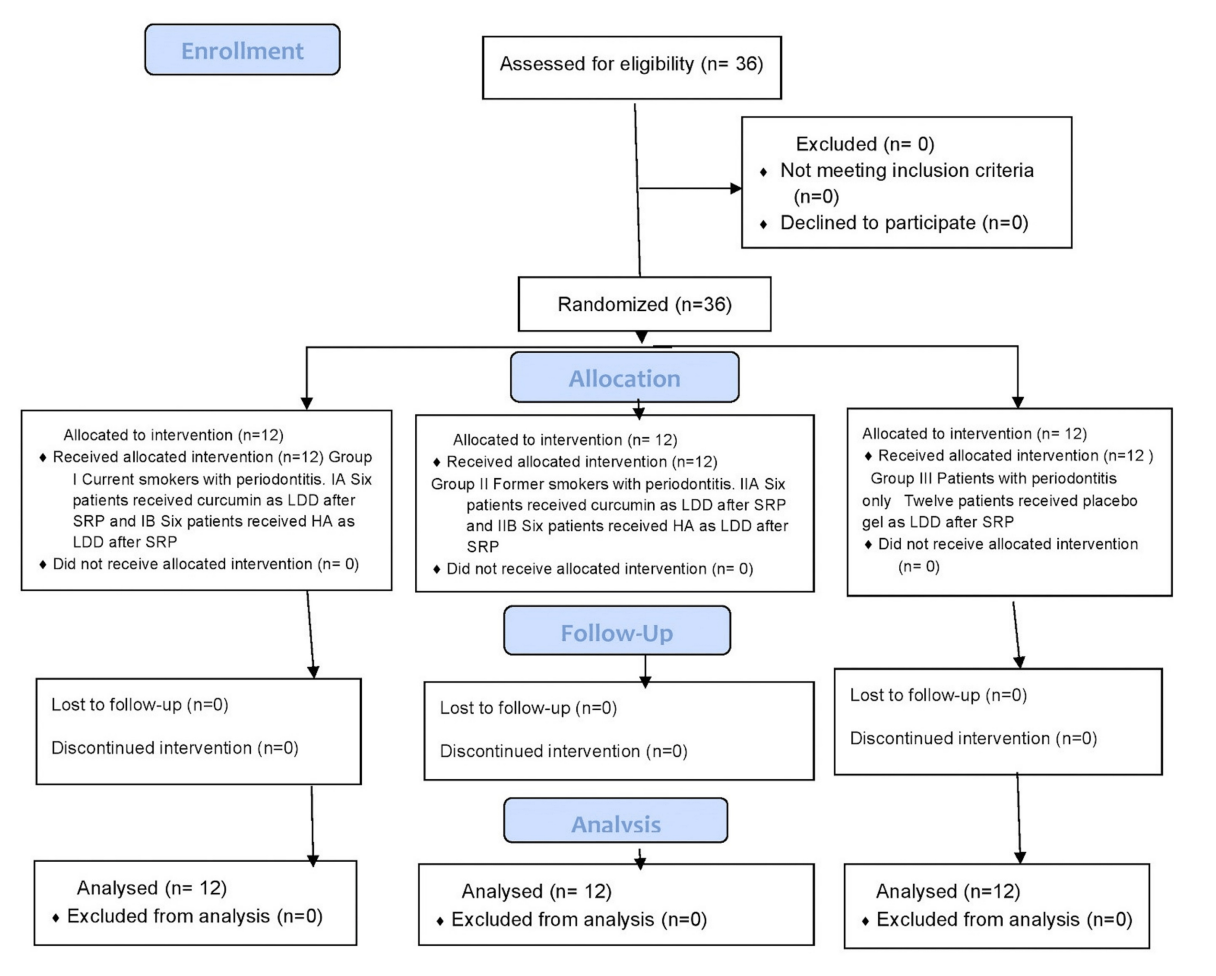
Consolidated Standards of Reporting Trials (CONSORT) flow diagram SRP: scaling and root planing, HA: hyaluronic acid, LDD: local drug delivery

Written informed consent was taken from all the patients participating in this study. The study was a prospective, single-blinded, interventional randomised trial that was approved by the institutional ethical committee (PMVIDS&RC IEC PERIO/DN329-20) and was also registered in Clinical Trials Registry-India (CTRI; CTRI/2021/05/033333) (Figure 1).

**Figure 2:**
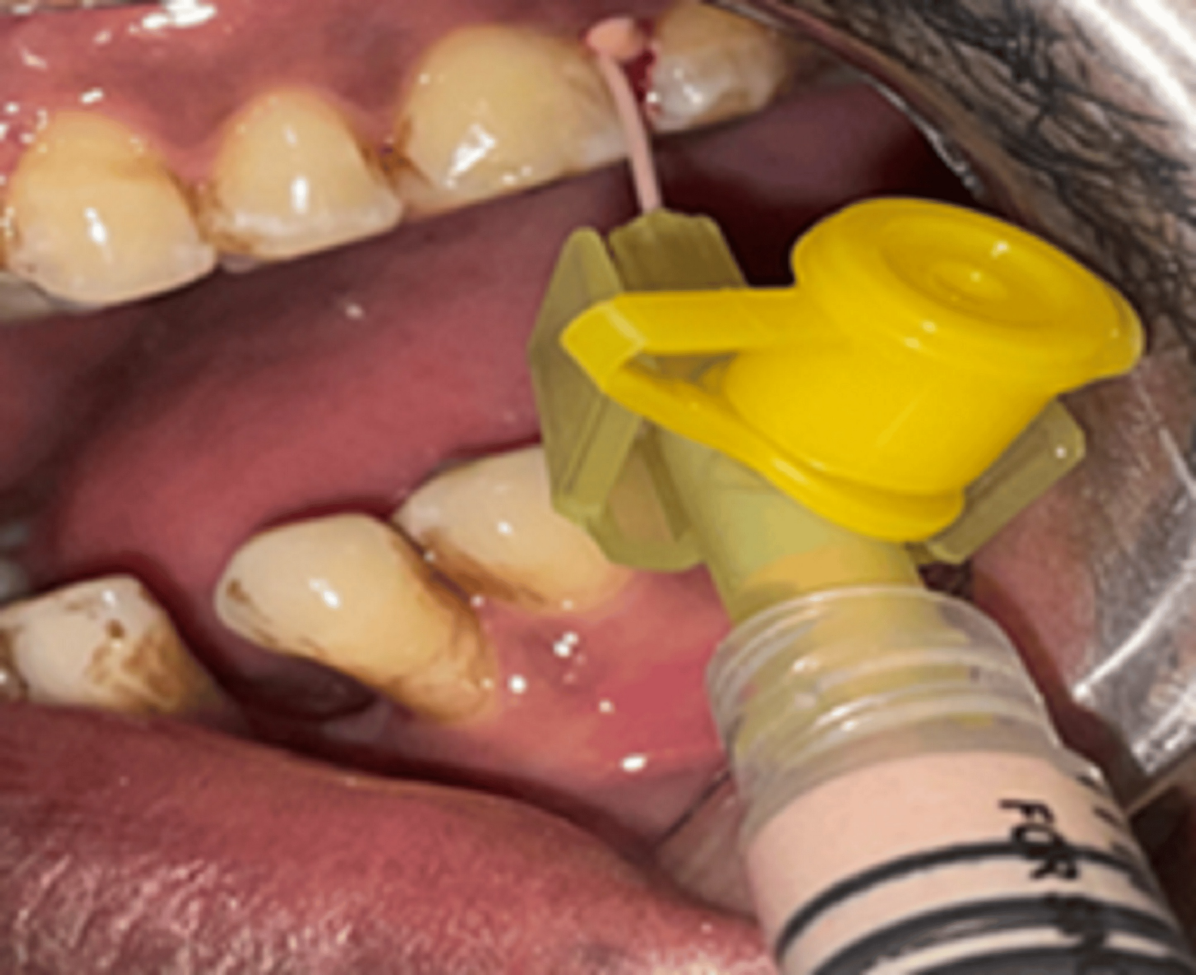
Group IA Curcumin gel was administered as local drug delivery after scaling and root planing (SRP)

**Figure 3:**
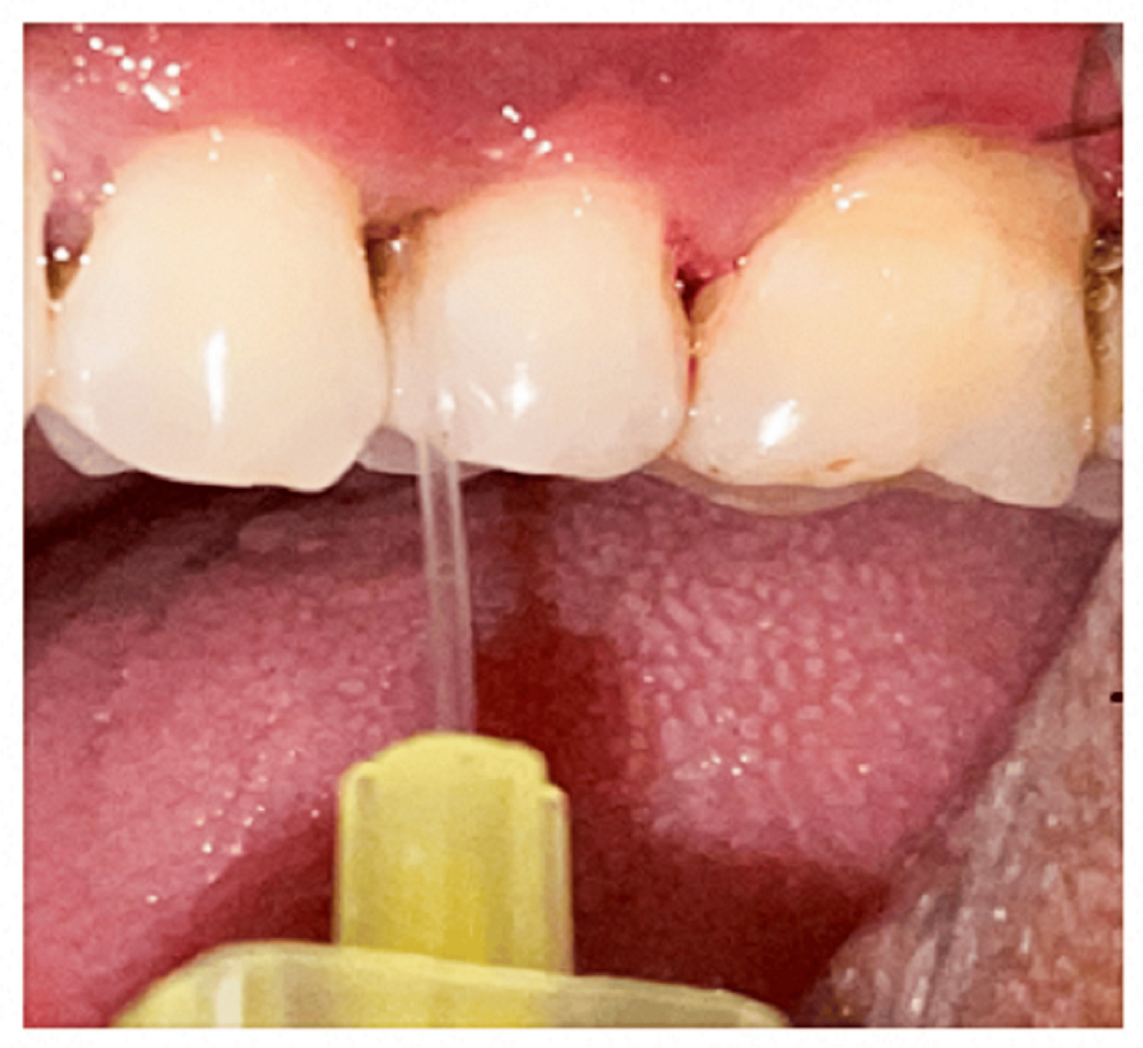
Group IB Hyaluronan gel was administered as the local drug delivery after scaling and root planing (SRP)

Related to their smoking history, the study samples were distributed into three groups. Group I included current smokers with chronic periodontitis (n=12) who underwent SRP, followed by local drug delivery of curcumin gel in subgroup IA (n=6) and hyaluronan gel in subgroup IB (n=6) (Figures 2, 3).

Group II included former smokers with chronic periodontitis (n=12) who underwent SRP, followed by local drug delivery of curcumin gel in subgroup IIA (n=6) and hyaluronan gel in subgroup IIB (n=6). Group III comprised patients with chronic periodontitis only, (n=12) who received SRP, followed by local delivery of placebo gel formulated with tragacanth gum and saline.

Inclusion criteria

For current smokers (Group IA & IB), those who smoked five or more cigarettes/day for the past one month were included. For former smokers (Group IIA & IIB), those who smoked five or more cigarettes/day and had quit the habit for the past one month were included. All the patients should have 20 teeth remaining and probing pocket depths of ≥5mm in one or more sites to be included in the study after periodontal examination.

Exclusion criteria

Patients with systemic diseases or autoimmune conditions, nursing mothers, pregnant women, those who had undergone periodontal treatment six months prior, and those taking medications were excluded.

Local drug delivery administration 

Initially a full-mouth SRP was performed, and all the participants were motivated to take care of their oral health. LDD was done using a blunt cannula syringe (2.5ml) injecting curcumin gel (10mg of Curcuma longa extract manufactured by Abbott Healthcare Pvt. Ltd., Bhiwandi, India), HA gel (0.2% gel manufactured by Ricerfarma, Milan, Italy), or placebo gel into the sulcus, followed by the placement of periodontal dressing. Patients were cautioned not to disturb the treated sites for one week. They were followed up after one week to rule out any adverse reaction to the LDD. The variables were recorded at the stipulated time periods.

**Figure 4:**
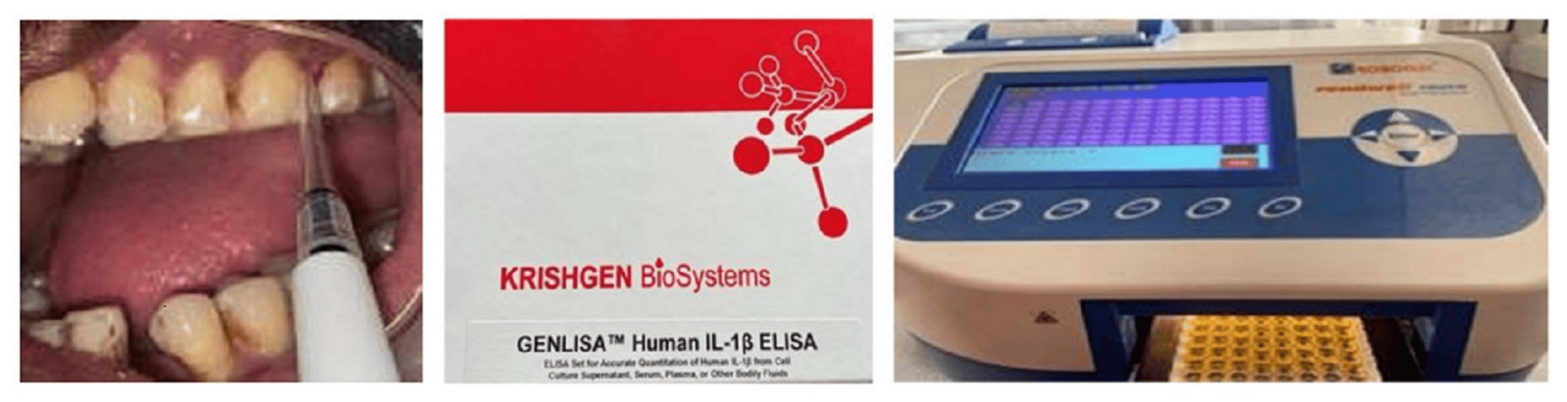
IL-1β estimation The gingival crevicular fluid (GCF) sample was analysed using the test kit for IL-1β by enzyme-linked immunosorbent assay (ELISA). A: GCF collection. B: GENLISA Human IL-Iβ ELISA Kit (Krishgen Biosystems, Mumbai, India). C: ELISA plate reader.

Biochemical parameters included IL-1β levels that were estimated using gingival crevicular fluid (GCF) as a source. The samples for IL-1β levels were collected at baseline and six weeks after nonsurgical periodontal therapy. Micropipettes were used for sample collection, and the samples collected (2μl of GCF) were diluted with phosphate buffer solution and stored at -20 degrees until the time of assay. An enzyme-linked immunosorbent assay (ELISA) was performed at baseline and six weeks postoperatively to read the samples (Figure 4).

**Figure 5:**
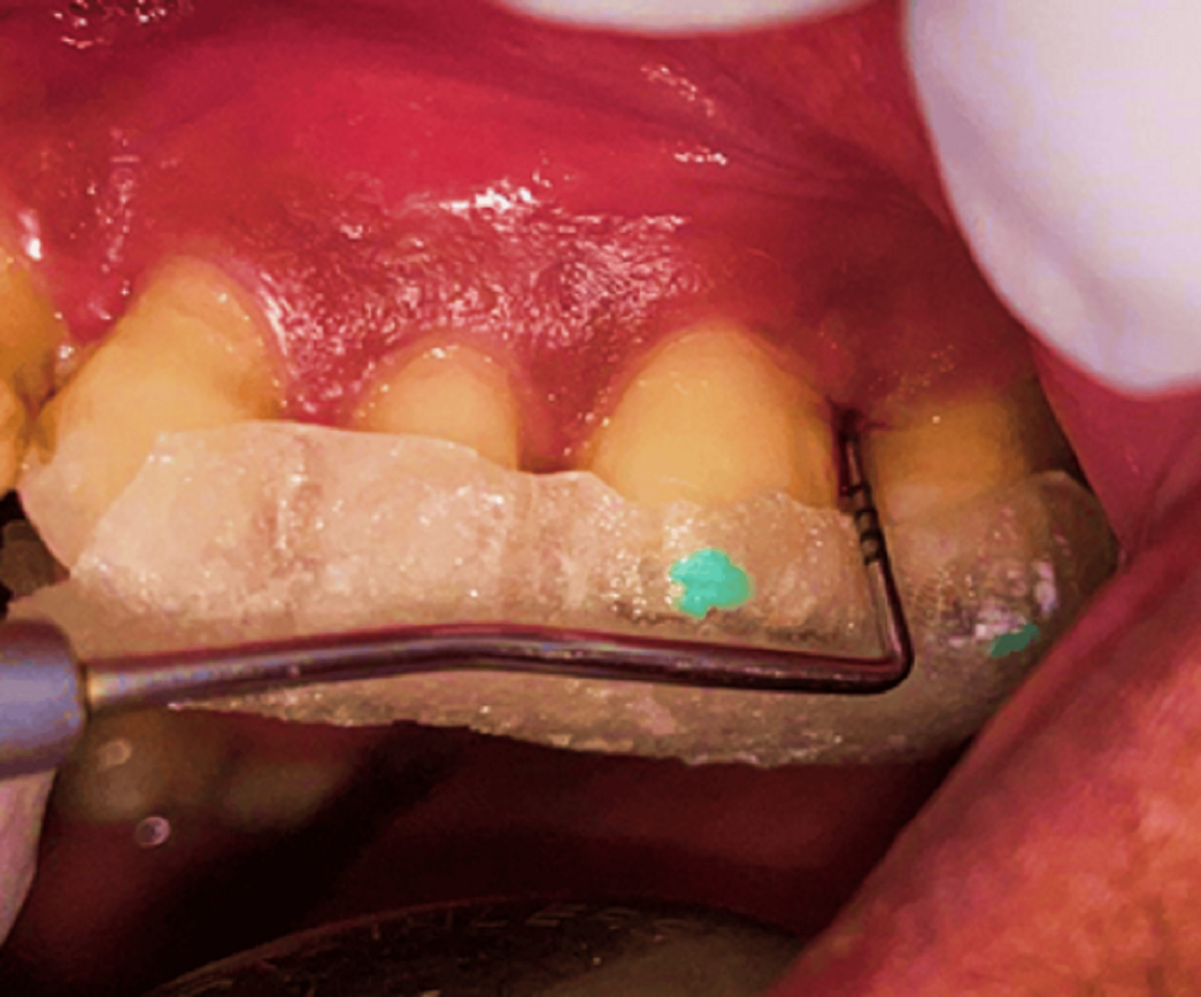
Assessment of clinical parameters A William's probe was used to evaluate the clinical parameters

Clinical parameters of Plaque Index (PI) and pocket depth (PD) (secondary outcome measures) were assessed at baseline, eight weeks, and 12 weeks after SRP using a William's probe (Figure 5).

## Results

PI evaluation

**Figure 6:**
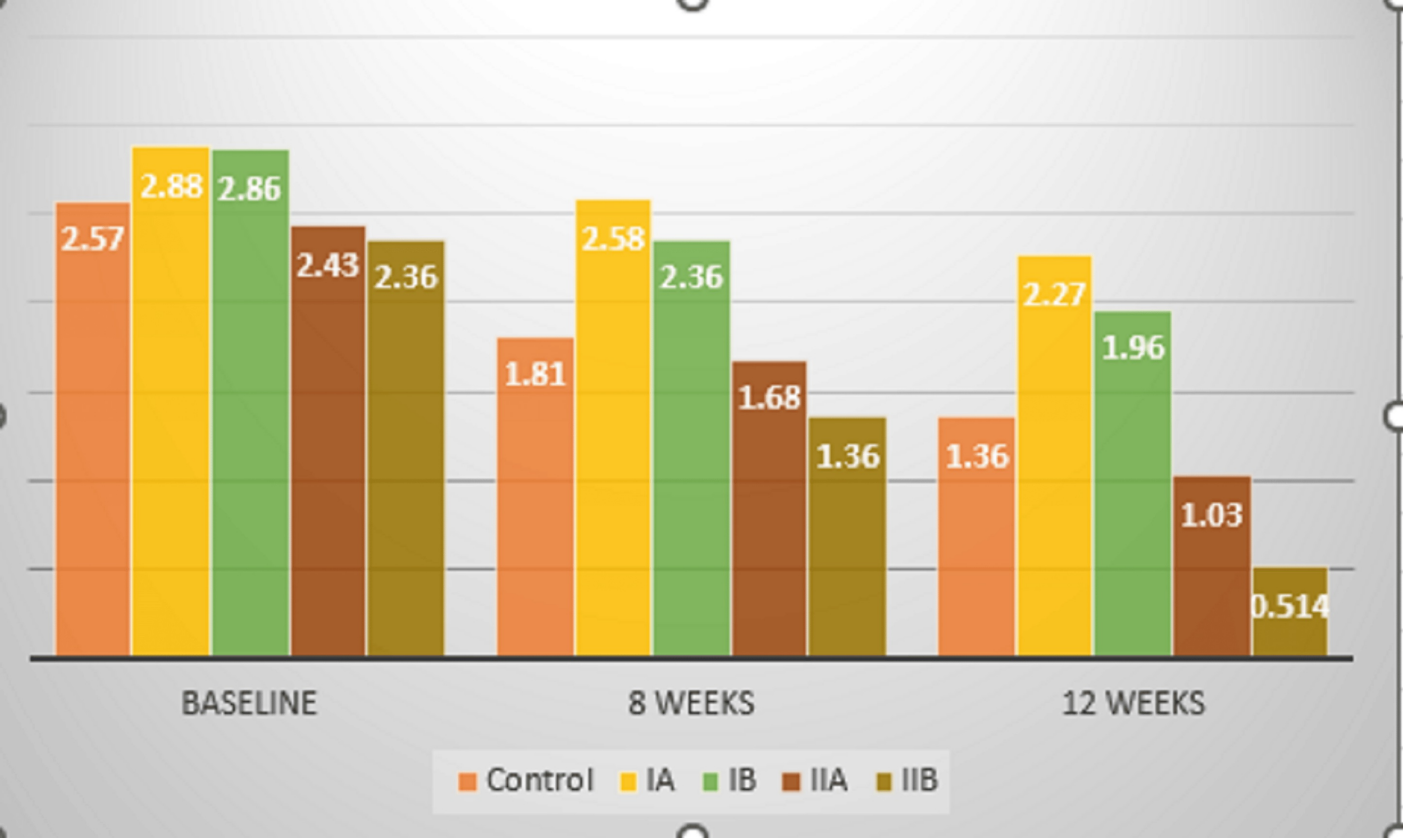
Mean comparison of Plaque Index (PI) levels between groups

The PI levels decreased in all the groups from baseline to eight weeks and 12 weeks. However, significant improvements were observed in Group II B (the PI levels were 2.36 at baseline, 1.36 at eight weeks, and 0.514 at 12 weeks). In Group II A, the levels were 2.43 at baseline, 1.68 at eight weeks, and 1.03 at 12 weeks. In Group III (control), the levels were 2.57 at baseline, 1.81 at eight weeks, and 1.36 at 12 weeks. Significant improvement was observed in Groups II A and B (former smokers), followed by Group III (control) (Figure 6).

PD evaluation

**Figure 7:**
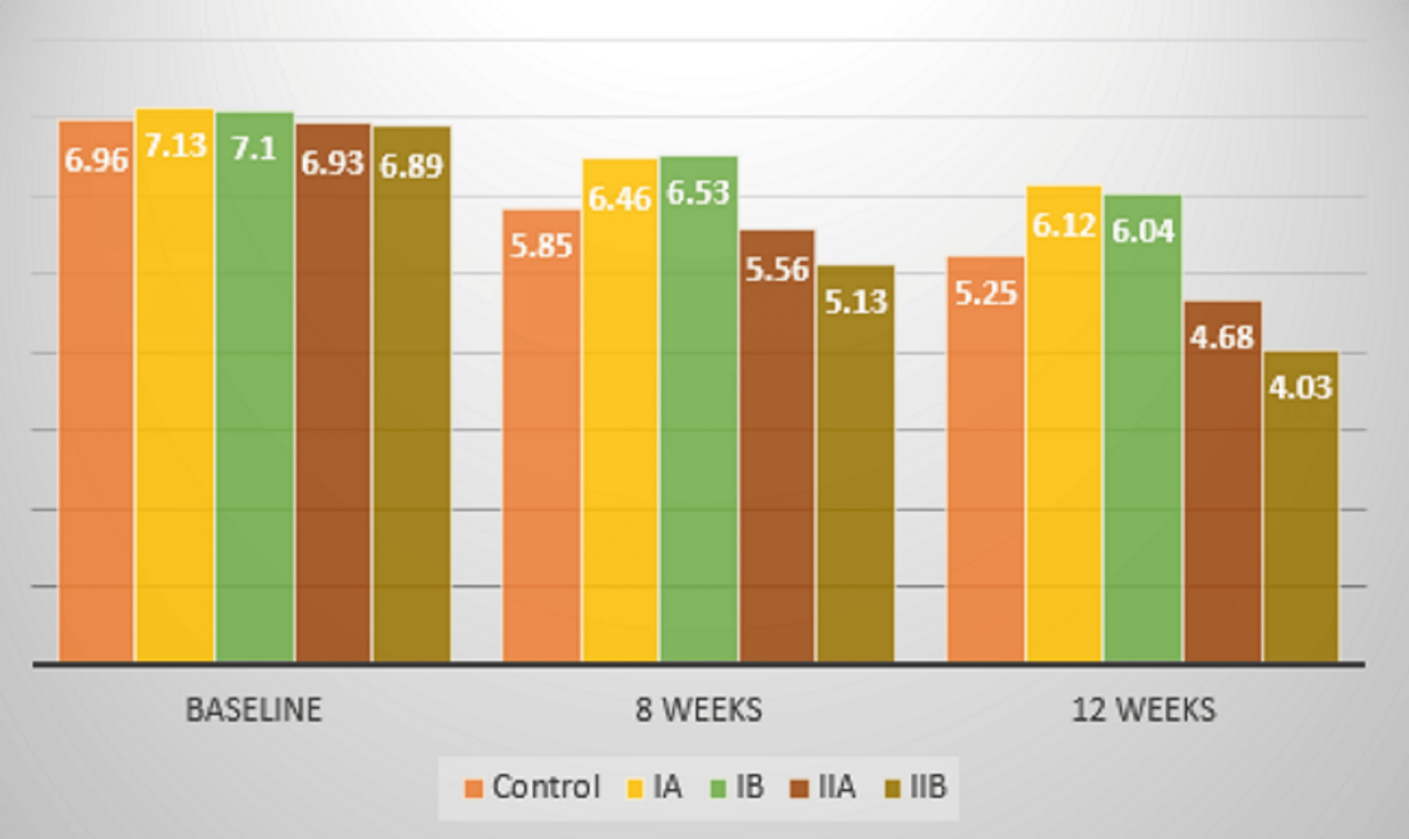
Mean comparison of pocket depth (PD) levels between groups

The PD levels decreased in all the groups from baseline to eight weeks and 12 weeks. However, significant improvements were observed in Group II B, wherein the PD levels were 6.89 at baseline, 5.13 at eight weeks, and 4.03 at 12 weeks. In Group II A, the levels were 6.93 at baseline, 5.56 at eight weeks, and 4.68 at 12 weeks. In Group III (control), the levels were 6.96 at baseline, 5.85 at eight weeks, and 5.25 at 12 weeks. Significant improvement was observed in Groups II A and B (former smokers), followed by Group III (control) (Figure 7).

IL1β evaluation

**Figure 8:**
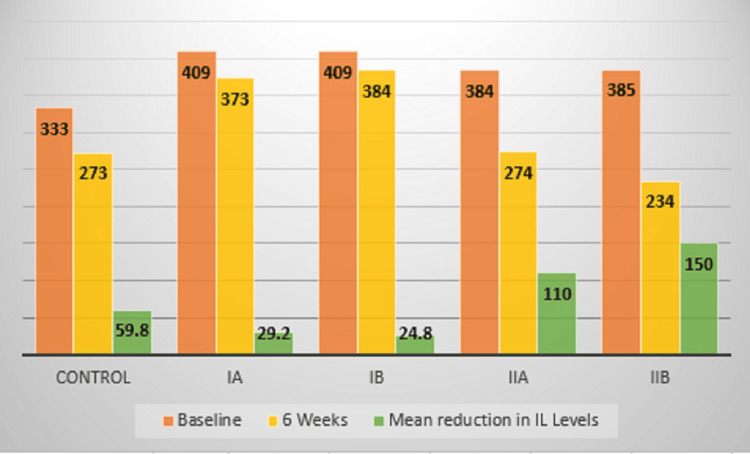
Mean comparison of interleukin 1β (IL-1β) levels between groups

The IL1β levels also significantly decreased from baseline to six weeks in all the groups. However, the reduction was significantly seen in Group II B, wherein, the levels reduced from 385 at baseline to 234 at six weeks with a mean reduction of IL1β being 150. In Group II A, the levels were 384 at baseline and 274 at six weeks, with a mean reduction of IL1β being 110. In Group III (control), the levels were 333 at baseline, 273 at six weeks, with a mean reduction of IL1β being 59.8. Significant improvement was observed in Groups II A and B (former smokers), followed by Group III (control) (Figure 8).

## Discussion

A putative microbe-laden biofilm is the harbinger of periodontitis, and smoking is a modifiable risk factor that affects the disease progression and treatment outcomes [6]. Many studies have stated that smoking is causally related to periodontitis, making it even more important to educate the public about smoking cessation protocols [7-9]. The levels of cytokines like IL1β in the saliva and GCF of smokers with periodontitis have been assessed in studies with contrasting results. A systematic review and meta-analysis comprising 17 studies conducted by researchers reiterated that smokers who underwent SRP did not show any improvement in clinical parameters like PD and clinical attachment (CAL) [10]. Results have been inconclusive when systemic antimicrobials have been used as adjuncts to nonsurgical periodontal therapy. To overcome the limitation of drug resistance, LDD systems have been used after SRP. Curcumin is one of the most extensively studied nutraceuticals, which has been approved by the FDA. Degradation of curcumin follows first-order kinetics [11]. Studies have been conducted using curcumin as an LDD agent in patients with periodontitis. Some researchers conducted a study to evaluate the effect of curcumin and ornidazole in treating chronic periodontitis. It was a split-mouth study wherein 20 patients having pocket depths >5mm bilaterally participated. After initial therapy, both gels were placed into the periodontal pockets on symmetric quadrants. PI, PD, and CAL were evaluated at days 0 and 30. At one month post-intervention it was observed that the clinical parameters significantly improved in the curcumin group over the ornidazole group. The authors stated that curcumin can be used as an adjunctive tool after SRP in patients with periodontitis [12]. Another study conducted on 10 patients assessed the efficacy of curcumin as an adjunctive tool after SRP on PI, gingival index (GI), PD, and CAL. This study also followed the split-mouth design, with the placement of curcumin as an LDD in two sites that had probing depths >5mm, whereas only SRP was performed in the contralateral quadrant. The PI and PD improved in the test group, whereas no significant improvement in CAL was observed [13]. There is very limited evidence to evaluate the efficacy of curcumin as an LDD system in smokers with chronic periodontitis. In the present study, curcumin gel showed a significant improvement in probing pocket depth in the former smoker group (Group II), in comparison to the current smoker group (Group I) (Figures 6, 7).

Hyaluronan, a non-sulphated glycosaminoglycan, is widely distributed throughout connective tissue, epithelial, and neural tissues. It is a critical component of the ground substance and contributes significantly to tissue hydrodynamics, cell migration, and proliferation. Hyaluronan is also produced by fibroblasts, and it inhibits tissue destruction and facilitates healing, so it plays an important anti-inflammatory role [14]. This study utilised 0.2% HA. Many studies have been done using this agent, and a study was conducted to evaluate the clinical effectiveness of adjunctive HA application to SRP on periodontal parameters and GCF volume in smokers with stage III and grade C periodontitis. Twenty patients participated in the study and were equally divided into the test (SRP+HA gel) and control (SRP only). It was deciphered that HA, as an adjunctive tool, did not show superior results to SRP alone [15]. Another study was conducted to assess the effects of adjunctive use of HA, clinically and microbiologically in smokers and non-smokers, with chronic periodontitis. The patients who participated included 12 smokers with periodontitis and 12 patients with periodontitis only. Two sites with PD >5mm were selected in each patient, with one site receiving HA gel and the contralateral site receiving a placebo gel. The PI, gingival bleeding scores, CAL, and PD were assessed at baseline, one month, and three months, whereas the microbiological parameters were evaluated at baseline and one month. The PD and CAL improved in both groups after three months, however, the results were better in the test sites (SRP+HA gel) over the control (SRP+placebo gel). The microbiological analysis showed a significant reduction in Aggregatibacter Actinomycetemcommitans and Porphyromonas gingivalis at the test sites when compared to the controls in both groups [16]. Even in this study it was observed that improved outcomes related to clinical parameters were observed in the former smoker group (Group II), compared to the current smokers (Group I) (Figures 6, 7).

Placebo gel (gum tragacanth or xanthan) was used as an LDD in this study, in the control group. Xanthan is a water-soluble mixture of polysaccharides obtained from sap that is drained from the root of the plant and dried [17]. The improvement in clinical parameters was found to be better in the control compared to the current smokers group (Group I).

IL-1β has been identified in the pathogenesis of periodontal destruction, it was formerly referred to as osteoclast activating factor and acts as a local mediator of tissue destruction in human periodontitis by inhibition of bone formation, stimulation of prostaglandin and thromboxane synthesis, collagenase and protease production, potentiation of neutrophil degranulation and superoxide production, enhancement of endothelial cell-leukocyte adhesion and stimulation of fibroblast and keratinocyte proliferation [18]. The GCF levels of IL-Iβ and IL-8 were assessed in smokers in yet another study. A total of 30 periodontitis patients, compartmentalised into smokers and nonsmokers, participated in this study. Clinical parameters assessed were GI, bleeding on probing (BOP), PD, and CAL. One diseased and one healthy site from each of the patients were selected for GCF collection and assigned to both the healthy and diseased sites in smokers and nonsmokers. ELISA was used for analysis, and it was concluded that smokers had elevated levels of IL-1β and reduced IL-8 levels, which reflects on the role of nicotine in the suppression of the immune system as well as in the progression of periodontitis [19].

Another study assessed the impact of SRP on GCF cytokine/chemokine levels in smokers with chronic periodontitis. Thirteen studies were included that were scanned from September 2017. The results pointed out that there was no change in the levels of the majority of the cytokines after SRP [20]. The results of this study were in accordance with the previous study, as it was observed that the IL1β levels in the GCF did not improve after SRP in the current smoker group (Group I), though improvement was seen in the former smokers and control group (Figure 8).

Limitations of the study 

The duration of drug release for curcumin gel and HA gel was not been assessed.

## Conclusions

Smoking is a modifiable risk factor for periodontitis. Smokers have suboptimal healing due to an impaired immune response. It has been observed that the outcome following nonsurgical and surgical periodontal therapy has been unfavourable in smokers. This study assessed the benefits of using curcumin gel and HA gel as LDD agents in both test groups after SRP. Though both agents had beneficial effects (with HA proving to be better), their benefits were appreciated only in the former smoker group, reiterating the importance of smoking cessation to help prevent periodontitis and improve the quality of life.
